# Dynamics and stability in prebiotic information integration: an RNA World model from first principles

**DOI:** 10.1038/s41598-019-56986-8

**Published:** 2020-01-09

**Authors:** András Szilágyi, Balázs Könnyű, Tamás Czárán

**Affiliations:** 1 Evolutionary Systems Research Group, MTA Centre for Ecological Research, Klebelsberg K. u 3, 8327 Tihany, Hungary; 2 MTA–ELTE Theoretical Biology and Evolutionary Ecology Research Group, Eötvös Loránd University, Pázmány P. s. 1C, 1117 Budapest, Hungary; 3 Center for Conceptual Foundation of Science, Parmenides Foundation, Kirchplatz 1, 82049 Pullach/Munich, Germany; 4 Department of Plant Systematics, Ecology and Theoretical Biology, Institute of Biology, Eötvös Loránd University, Pázmány P. s. 1 C, 1117 Budapest, Hungary; 5 Biocomplexity Group, Niels Bohr Institute, Copenhagen University, Blegdamsvej 17, 2100 Copenhagen, Denmark

**Keywords:** Systems biology, Computer modelling, Dynamical systems, Evolvability, Population dynamics, Ecology, Ecological modelling, Evolutionary ecology, Evolution, Origin of life

## Abstract

The robust coevolution of catalytically active, metabolically cooperating prebiotic RNA replicators were investigated using an RNA World model of the origin of life based on physically and chemically plausible first principles. The Metabolically Coupled Replicator System assumes RNA replicators to supply metabolically essential catalytic activities indispensable to produce nucleotide monomers for their own template replication. Using external chemicals as the resource and the necessary ribozyme activities, Watson-Crick type replication produces complementary strands burdened by high-rate point mutations (insertions, deletions, substitutions). Metabolic ribozyme activities, replicabilities and decay rates are assigned to certain sequence and/or folding (thermodynamical) properties of single-stranded RNA molecules. Short and loosely folded sequences are given replication advantage, longer and tightly folded ones are better metabolic ribozymes and more resistant to hydrolytic decay. We show that the surface-bound MCRS evolves stable and metabolically functional communities of replicators of almost equal lengths, replicabilities and ribozyme activities. Being highly resistant to the invasion of parasitic (non-functional) replicators, it is also stable in the evolutionary sense. The template replication mechanism selects for catalytic “promiscuity”: the two (complementary) strands of the same evolved replicator will often carry more than a single catalytically active motif, thus maximizing functionality in a minimum of genetic information.

## Introduction

Copying processes, whether natural or artificial, are always prone to errors. The unavoidably high error rate of non-enzymatic RNA replication is the central problem of the *RNA world scenario*^1,2^ of the origin of life, which is still the most plausible paradigm for research on prebiotic evolution. The problem itself is of the chicken-or-egg type: the first RNA sequences presumably supplied by physico-chemical processes on prebiotic Earth were far too short to code for the catalytic agents of even an inefficient self-replication mechanism, the absolute minimum requirement for the evolutionary improvement of replicase functionality to set in at all (Eigen’s paradox^3^). The mathematical relation between the amount of chemically encoded information sustainable through many cycles of replication and the highest tolerable mutation rate thereof, called the “error threshold”, was derived by Eigen and Schuster in the 1970s^4^, but a feasible chemical scenario providing a plausible resolution to Eigen’s paradox was not available then.

The various attempts made in the last 40–50 years to resolve the paradox fall into two broad categories assuming radically different evolutionary mechanisms to operate:

(1) gradually increasing the length of the self-replicating replicator and its copying fidelity at the same time (the sequence elongation or “bootstrap” approach^5–7^), and

(2) ensuring the stable coexistence of short replicators whose collective information content exceeds the minimum needed to encode the replicase (the ecological approach^7–9^).

For a recent review on the ecological and evolutionary stability and the diversity-maintaining ability of different bootstrap models of prebiotic evolution^10^. Approaches based on the effect of lethal and back mutations^11^, phenotypic (rather than genotypic) preservation of catalytic activity^12^, or wobbling in the genotype-phenotype projection^6^ have been proposed as possible ways to achieve error thresholds higher than predicted by Eigen’s calculation. Elegant and revealing as they are, these mechanisms cannot provide sufficiently effective ways of transferring a necessary minimum amount of information from generation to generation. Even less sufficient they are to run the simplest of proto-cells, because a single RNA molecule may have a very limited number of catalytic activities at best, definitely not appropriate to run even the simplest of metabolisms (cf. studies on minimal genomes^13^). The plausible way to keep sufficient genetic information present and functional in an RNA world setting seems to be the maintenance of communities of shorter sequences, each individual replicator shorter than the maximum set by the error threshold, but their collective information content sufficient for coding a self-replicating system. Such a replicator community inevitably had to face all the ecological problems related to the coexistence of competing entities.

**Figure 1:**
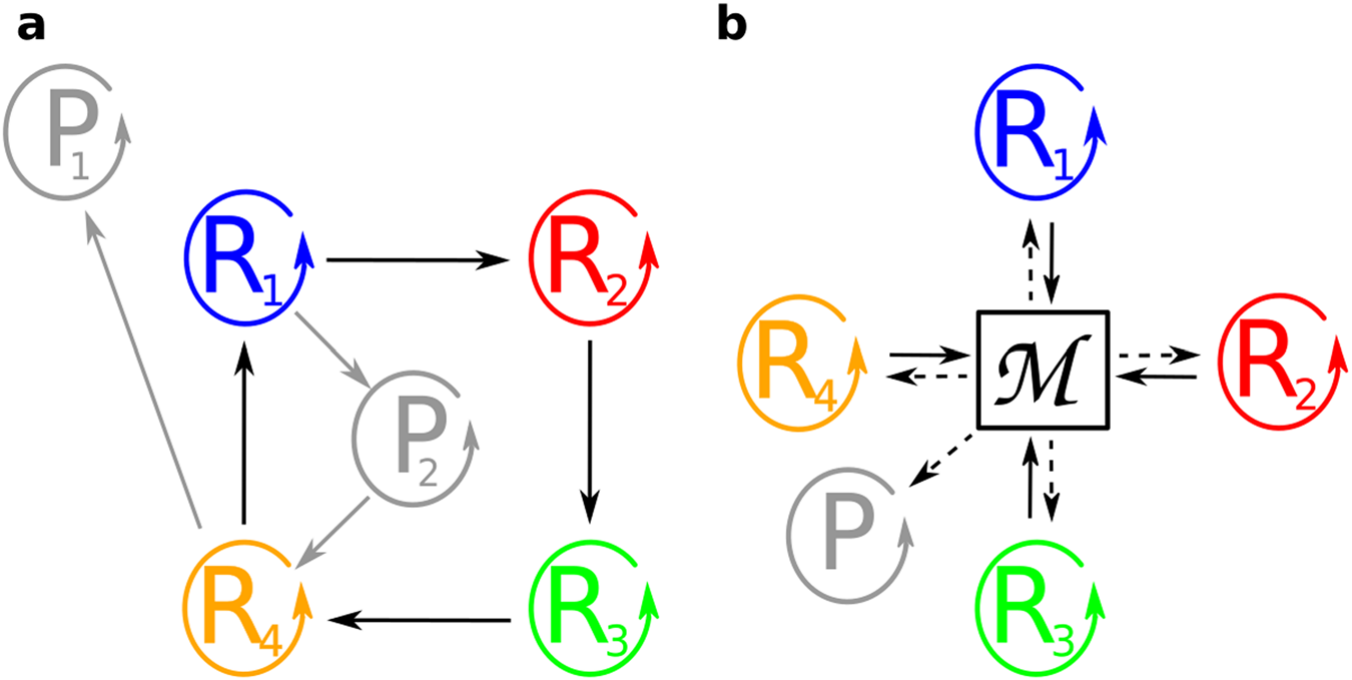
Metabolically coupled prebiotic replicator communities. **(a)** Schematic representation of the catalytic interactions in a four-membered hypercycle of Eigen and Schuster. Solid arrows symbolize catalytic aid. **(b)** Schematic representation of the catalytic interactions in a four-membered Metabolically Coupled Replicator System (MCRS). Each replicator catalyses a single reaction (solid arrows) of a metabolic network (*M*) producing the monomers that all the replicators are built from (dashed arrows).

A straightforward method of keeping different replicators permanently coexistent in an ecological equilibrium state is to make their persistence dependent on each other, for example by heterocatalytic aid for replication given and received between the replicators in a circular topology: this is the core idea of the hypercycle of Eigen and Schuster^4^ (Fig. 1a). The community of short replicators collectively encodes the total information content of the system. Unfortunately, the hypercycle is not robust in the topological sense: selfish and shortcut mutants (parasites) of the hypercycle may gradually eliminate all the links of cooperation and thus destroy the community^14^. It is also unstable in the ecological sense: mutants preferentially helping any replicator that is not a member of the hypercycle will pump out resources from the community and thus destroy it.

The collapse of the community can be avoided by having the replicators cooperate in a constitutive manner through a non-cyclical system of indirect and obligate mutualistic interactions. This is the core assumption of the Metabolically Coupled Replicator System^15^ (MCRS) in which all enzymatically active replicator types contribute to a common metabolism producing the monomers that all the replicators are built from (Fig. 1b). The contribution of each replicator to the metabolic reaction network through its specific enzymatic activity is essential for the system to persist: any one of them missing breaks down monomer production and thus also the replication process. This means that the cooperative (mutualistic) interaction within the community of replicators is obligatory.

The dynamics of the hypercycle and the MCRS are fundamentally different. The hypercycle is coexistent within a well-mixed, homogeneous system until parasitic mutants (selfish or shortcut parasites) destroy it^14^, whereas the MCRS requires spatial structure (on mineral surfaces or in membrane compartments) to survive at all^15,16^, but the spatial MCRS is also resistant to parasitic replicators, effectively keeping them at low frequencies within the replicator community even if the parasites have a substantial edge compared to cooperating replicators in terms of their dynamical parameters^15^. The parasites of the MCRS are replicators using the monomers but failing to produce them – these are free to mutate to any length and replication rate. The group selection mechanism of the system keeps eliminating these parasites and maintaining the cooperation of the metabolically active community.

In a series of earlier studies^8^ we have shown that the surface-bound MCRS can maintain the robust coexistence of replicator communities and resistant to mass invasions by its parasites (*stability*). It is also inherently pre-adapted to acquiring new metabolic (or, in fact, any other useful) functions by potentially converting randomly mutating parasites into enzymatically active replicators – another beneficial feature (*evolvability*) that is practically impossible to imagine to occur in the hypercycle^8,17,18^. The MCRS is capable of domesticating mutant replicators with new catalytic activities, and thus of expanding the network of metabolic reactions in accordance with the “retroevolution” scenario^8,19^. However, the maximum size of a metabolically complete replicator community (i.e., the number of different replicators that are indispensable for running metabolism) remains limited^20^. Thus the number of enzyme activities that metabolism may adopt is also limited. One possible escape from this limitation is assuming “catalytic promiscuity”^21,22^, meaning that some (or all) of the replicators may accommodate more than a single enzyme activity, which would be fostered by at least two strong selective pressures. One of these is the advantage of packing as many phenotypic functions into as little genetic material as possible, the other the selective advantage of chromosomization: the optimized dosage of phenotype functionality in a single sequence.

In this paper, we explore the ecological and evolutionary consequences of template replication^23^ and catalytic promiscuity in the MCRS model, assuming explicit primary structures (nucleotide sequences) and the corresponding 2D secondary structures (calculated by the ViennaRNA algorithm^24^) of both complementary strands of every replicator in the system. Primary and secondary structures along with the corresponding folding energy determine the actual replicability, decay rate and catalytic activity of each sequence. This means that the actual dynamical properties of the replicators are directly derived from first physical principles in as much detail as recent computational limitations permit. We will show how the template mechanism of replication affects the ecological properties (stability and robustness of coexistence) and the structural composition of the evolving replicator community^23,25^. We will also investigate a possible route to the emergence of two types of enzymatic promiscuity (*cis* and *trans*, i.e. same-strand and complementary-strand catalytic multiplicity, and derive Eigen’s well known “error threshold” in the context of the MCRS.

## Materials and Methods

Here we briefly summarize the characteristic features of the MCRS model with explicit replicator folding and sequence complementarity.

### Metabolism and replication

**Figure 2:**
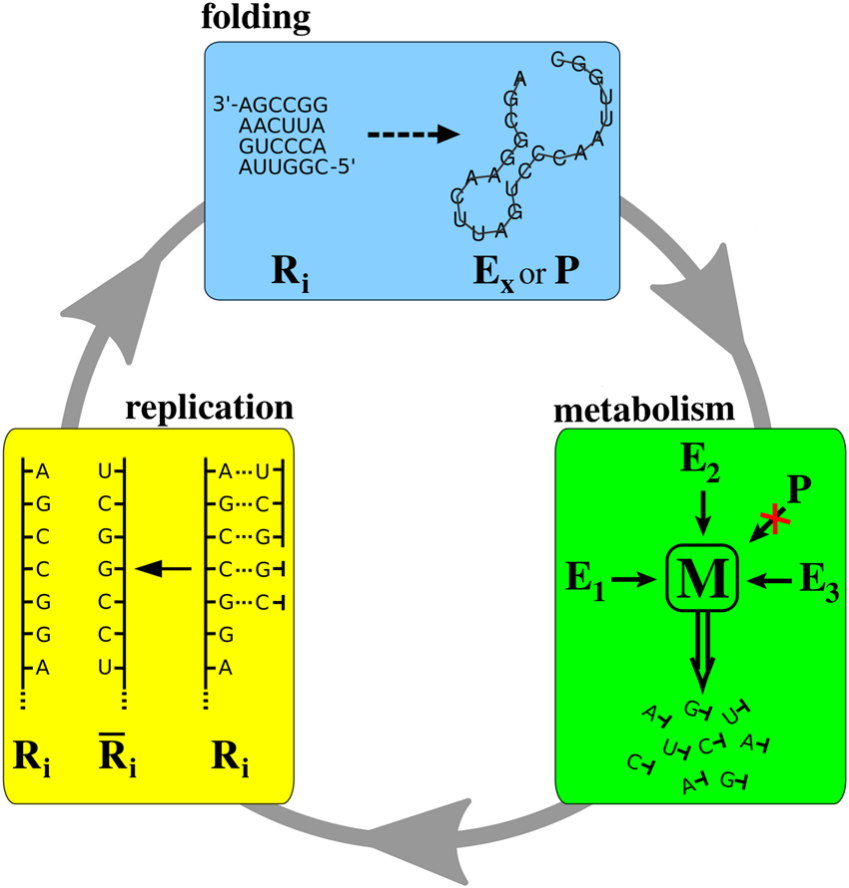
Main steps of MCRS model. The *folding* algorithm (ViennaRNA) finds the secondary structure of lowest energy and the corresponding (Gibbs) free energy of the optimal folded state. *Metabolism* produces monomers if all the necessary enzymatic activities (*E*_1_, *E*_2_ and *E*_3_ in the figure) are present in the metabolic neighbourhood centred on the focal replicator (metabolic completeness). *Replication* produces the complementary copy of the focal template in accordance with the Watson-Crick rules of base pairing.

Technically, the Metabolically Coupled Replicator System model is a 2D cellular automata implemented on a square lattice of toroidal topology to avoid edge effects. The outer surface of the toroidal lattice represents the mineral surface which RNA replicators are reversibly attached to. The partially immobilized replicators interact with each other locally through their specific catalytic (ribozyme) activities, each of which is assigned to a specific loop structure and/or sequence motif, i.e., the catalytic functionality of a replicator is defined by its primary and secondary structure. Interaction means the collective production of monomers in compulsory metabolic cooperation: a local population of ribozyme replicators representing a pre-determined set of different catalytic activities has to be present within a certain area of the mineral surface (metabolic neighbourhood) for nucleotide monomers, the essential building blocks of RNA, to be produced in that area. Therefore, if any one of the essential catalytic activities is missing from the corresponding neighbourhood local RNA replication stops altogether due to lack of monomers. Metabolism is implicit, meaning that metabolite concentrations are not explicit variables of the model; monomers are assumed to be present at a concentration sufficient to accomplish replication within any complete metabolic neighbourhood. The size of metabolic neighbourhoods defines the spatial extent of the surface-bound diffusion of metabolites: at distances exceeding the radius of a metabolic neighbourhood the concentration of monomers produced within that neighbourhood and still attached to the surface is assumed to be negligible. Metabolites drifting out of the neighbourhood they were produced in are lost to the bulk (dissociate from the surface into the third dimension), never to reattach to the surface and become available for replication again. RNA polynucleotides (both the template and its copy) can diffuse on the surface to some extent, and if they find themselves in a complete metabolic neighbourhood they can replicate again. Single base mutations (insertions, deletions, substitutions) occur at predefined rates during replication. Figure 2 summarizes the main components of the MCRS scenario. For a detailed description of the model assumptions, see Supplementary Information Section 1.

### Replicator properties

Replicators are RNA molecules represented by their nucleotide sequences, secondary structures and Gibbs free energy. These are the properties of a replicator which we consider pivotal, because primary and secondary structures are assumed to define the type of catalytic activity, and free energy determines the degradation rate of the RNA molecule besides its indirect effect on catalytic activity and replicability. The most stable 2D secondary structure and the corresponding free energy of each RNA molecule on the surface are computed by the ViennaRNA package^24^. Gibbs free energy (*E*) defines the probability that a sequence is in the folded state (*p*_fold_) according to the Boltzmann distribution: ${p}_{{\rm{fold}}}=\frac{{e}^{-cE}}{1+{e}^{-cE}},$ where *c* > 0 is a parameter. The lower the free energy the more stable the secondary structure, cf. Supplementary Equation (S1). Within the sequence length interval *L* = 15…75 used in the model, free energy has an empirical lower bound at ${E}_{{\rm{\min }}}=-\,25\,{\rm{kcal}}/{\rm{mol}}$. With applying *c* = 0.3, a sequence with about zero free energy is in the folded state with probability ½, while for a sequence with *E*_min_ (i.e., in an energetically optimal structure) the probability of being folded is almost 1.0. Free energy also defines the degradation rate (*p*_deg_) of a sequence: lower free energy states stabilize secondary structures and thus reduce degradation probability: ${p}_{{\rm{\deg }}}={\delta }_{0}-{\delta }_{1}\frac{E}{{E}_{{\rm{\min }}}}$, where δ_0_ is the invariant part and δ_1_ is the free energy proportionality factor (δ_0_ > δ_1_, to avoid “immortality” of replicators), see also Supplementary Equation (S3). (Note that the complications of strand separation after replication have been disregarded here, therefore the effect on the degradation rate of template and copy strands possibly sticking together is also neglected).

Metabolic activities are defined by the primary and secondary structures of the replicators: different activities correspond to different loops and sequence motifs. We assume that metabolism consists of a three-step network of metabolic reactions, each step catalysed by a different ribozyme. The activity of the first one requires the presence of a five base long sequence motif (which is about the length of the shortest known enzymatically active RNA sequence^26^) anywhere within the non-paired region of the folded RNA molecule (outside of loop necks and hairpins). The second and the third activity correspond to a smaller and a larger loop, respectively, with different, activity-specific, four base long motifs anywhere inside the loops. Sequence errors in specific motifs and/or differences in the length of loops decrease the specific activity α of the ribozyme according to the rules given in Supplementary Table S1. Since a ribozyme can act as an enzyme only in its folded state, the probability *p*_fold_ and the specific activity α jointly define the actual catalytic activity *a* of any sequence.

The template mechanism of replication ensures that RNA replicators exist in two complementary strands, each of which can accommodate one or more catalytically active sites. Any replicator with more than a single activity is called “*promiscuous*”. *Cis*-promiscuity occurs if one of the two complementary strands of a sequence is responsible for two or more enzymatic activities. This requires the sequence to be sufficiently long so that two (or) more loops and/or motifs fit on it. The activities of cis-promiscuous strands are lower than that of single-activity strands of the same active site sequence pattern because of steric reasons. Since a single strand of RNA cannot catalyse more than one reaction at a time, the time sharing between different types of catalysis apparently reduces all kinds of activities present on the same strand by a sub-additive factor *m*^σ^ (for details, see Supplementary Information Section 1.2). Thus, the actual activity of a catalytic site on a cis-promiscuous strand is *a* $=\,{p}_{{\rm{fold}}}\frac{\alpha }{{m}^{\sigma }}$ (see Supplementary Equation (S2) for details). The other type of promiscuity is *trans*-promiscuity: in this case one strand of the replicator catalyses one reaction and its complementary strand catalyses another one. *Trans*-promiscuity is not punished directly, but it may be the cause of a temporal disadvantage compared to cis-promiscuity: a trans-promiscuous ribozyme can catalyse only one reaction at any time (behaves as a specialist enzyme), but swaps activity in each generation^23^. Therefore, a trans-promiscuous ribozyme carrying a locally rare catalytic activity may provide the critical functionality in its own metabolic neighbourhood only in half of the cases.

### Replication

For a successful replication event in the metabolic neighbourhood of the focal replicator all the three types of enzymatic activities must be present, i.e. its neighbourhood must be metabolically complete. The *metabolic activity M*_*f*_ of the focal replicator *f* is the geometric mean of the essential enzyme activities within its metabolic neighbourhood Δ_*h*_(*f*): ${M}_{f}={[\mathop{\prod }\limits_{i=1}^{3}\sum _{j\in {\Delta }_{h}(f)}{a}_{i,j}]}^{\frac{1}{3}}$, where *i* runs over the three activities and *j* are the indices of sites in the metabolic neighbourhood Δ_*h*_ of *f*. In line with the underlying (bio)chemical assumption this choice guarantees that in the absence of one or more necessary activities the local monomer supply of the focal replicator, and thus the chance that it is replicated in the given time step, is zero. The geometric mean also favours the even distribution of different enzymatic functions within a metabolic neighbourhood. Replication is possible only if the replicator is in its unfolded state, thus its replicability is proportional to *l* + (1 − *p*_fold_), where *l* > 0 ensures that even replicators at the lowest energy state, *E*_min_, can replicate to some extent. We assume that the time required for a complete replication consists of two phases: the length of the first phase is proportional to the length *L* of the replicator (*b*_2_), whereas the length of the second (consisting of the initiating and terminating steps) is invariant (initiating and terminating step, factor *b*_1_). Thus, the replicability of the focal individual is ${R}_{f}=g\frac{l+(1-{p}_{{\rm{fold}}})}{{b}_{1}+{b}_{2}L}$ (where *g* is a constant parameter, see also Supplementary Equation (S5) for details). We define the *claim C*_*f*_ of replicator *f* belonging to the replication neighbourhood of an empty site to replicate into that empty site as the product of its metabolic activity *M*_*f*_, and replicability (*R*_*f*_) as *C*_*f*_ = *M*_*f*_ *R*_*f*_. The probability of the focal replicator *f* to replicate into an empty site in its replication neighbourhood is proportional to its own share of all claims within the replication neighbourhood to occupy the empty site: ${P}_{f}=\frac{{C}_{f}}{{C}_{e}+{\sum }_{j}{C}_{j}}$, where *j* runs across the replication neighbourhood of the empty site and *C*_*e*_ is the claim of an empty site to remain empty. During replication point mutation events (substitution, insertion and deletion) can occur with the per base probabilities *p*_sub_, *p*_ins_, and *p*_del_, respectively.

Note that the replicability – enzyme activity trade-off is an inherent and emergent property of the model introduced via the Gibbs free energy of the actual RNA strands. Low-energy sequences tend to be better and more durable ribozymes but more difficult to copy, whereas high-energy sequences are easier to replicate but are likely to be less efficient metabolically and more at risk of hydrolytic degradation.

### Dynamics of the system

The dynamics of the system is followed on a toroidal grid of 300 300 sites. The diffusive movement of the replicator molecules on the surface has been implemented with the Toffoli—Margolus algorithm^27^. The simulations were initiated with the spatially random allocation of a mixture of catalytically active and inactive sequences on the grid. The initial sequences were randomly assembled, but functional ribozymes were over-represented in the initial state to ensure that all viable systems take off (i.e., to avoid random extinction due to the limited number of replicators present in the initial state). Simulations with different proportions of catalytically active sequences in the initial replicator populations show no difference in the end states in the qualitative sense (for details, see Supplementary Information Section 1 and Supplementary Fig. S1). Starting from the augmented random initial distribution of replicators within the 300 300 square lattice the degradation probability *p*_*deg*_ and the replicability *R* are calculated for each replicator present in the lattice from its actual sequence at time *t*, using the ViennaRNA folding algorithm. The metabolic support *M* it receives is also calculated from the sequences of the replicators within its metabolic neighbourhood. Applying these individual sequence-dependent parameters the fate (degradation, survival, replication) of each replicator is determined. Replication produces the complementary copy of the template in accordance with the Watson-Crick rules of base pairing. The per-base mutation rate is applied for mis-pairing, nucleotide deletion or insertion at each step of the replication process. This updating algorithm is repeated at a random order to each site of the lattice once on average, resulting in 90.000 elementary updates (Supplementary Fig. S2) per unit time.

## Results

The parameters of highest dynamical impact in the sequence-explicit MCRS model are the diffusivity of the replicators (*D*) and the length-dependent component of replicability (“length penalty”, *b*_2_), cf. Fig. 3 of Könnyű *et al*.^28^. The model shows very weak sensitivity to the other parameters, and with the computing capacity constraints due to tens of billions of folding algorithm iterations per 2.5 million generations (the length of the simulation experiments) in mind we have run the model with just two different values for each of these critical parameters (*D* = 1.0 and 4.0, and *b*_2_ = 0.005 and 0.01) and five replicate runs in each of the four parameter combinations. We will refer to these parameters as high *D*, low *D* and high *b*, low *b*, respectively. These parameter combinations were used for the simulations, some of the results are shown in the main text, others can be found in the Supplementary Information. The fixed values of all other parameters are given in Supplementary Table S2.

**Figure 3:**
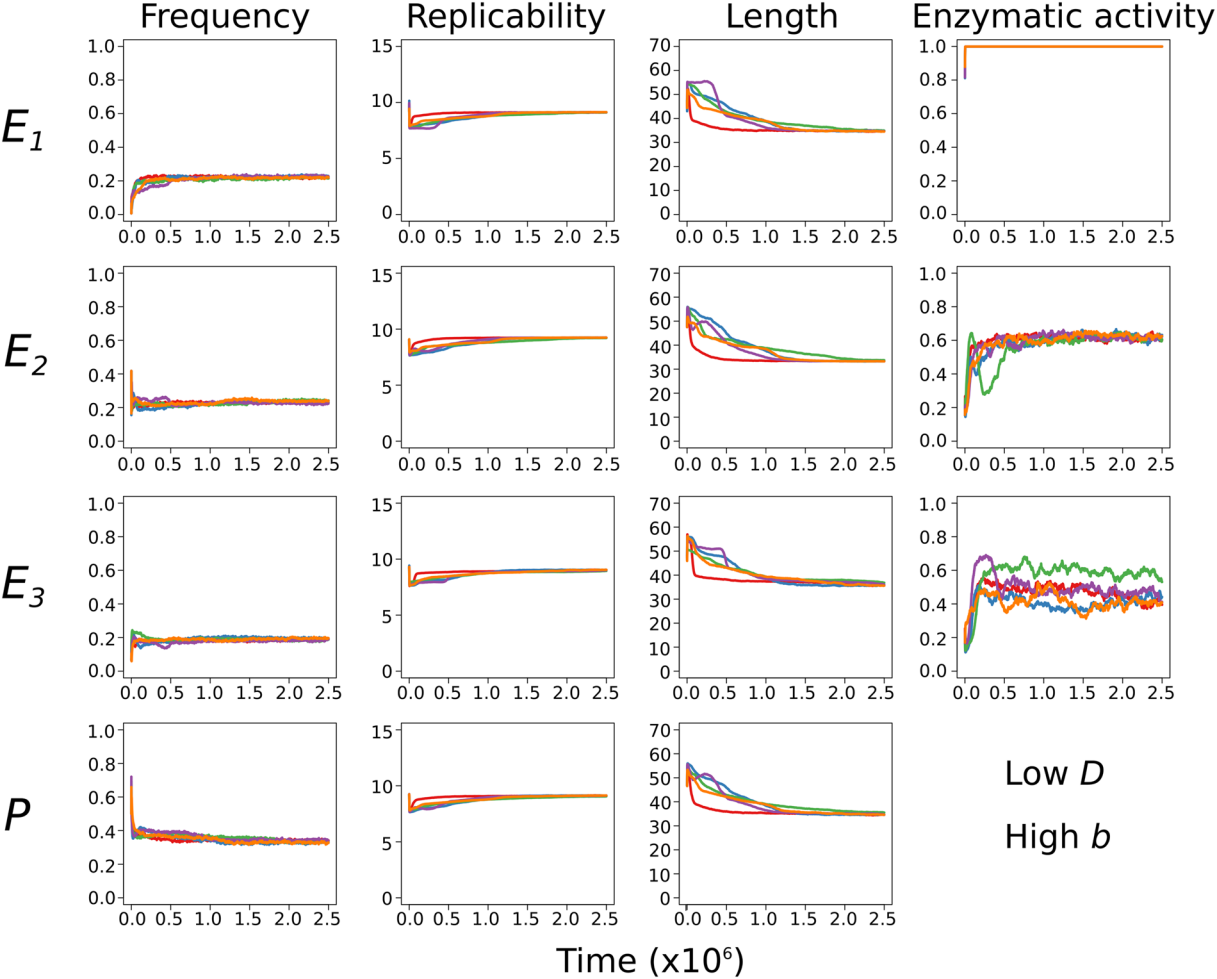
Time series from parallel simulations. Plots of five independent simulations, each in a different colour. First column: frequencies of the three monoactive enzymes (*E*_1_ – *E*_3_) and functionless (parasitic; *P*) replicators; second, third and fourth columns: average replicabilities, lengths and enzymatic activities (based on Supplementary Equation (S5)), respectively. Initial proportion of enzymatically active replicators: 50%. Parameters: *D* = 1.0, *b*_2_ = 0.01 (low *D* – high *b*).

A layered random initial distribution (details in Supplementary Information Section 1.4) of replicators was applied at a 50% frequency of metabolically active sequences with 80% of the sites occupied, resulting in 40% of the lattice harbouring metabolically active replicators at *t* = 0. As mentioned before, numerical experimentation revealed that this does not affect the qualitative outcome of the simulations.

### Ecology: steady states and stability

The ecological stability of the MCRS strongly supported by the fact the simulated dynamics of replicator properties (frequencies, average enzyme activities, average replicabilities, and average sequence lengths) converge. The equilibrium values of these properties depend on the actual set of parameters, but they are insensitive to initial conditions (ribozyme compositions and concentrations) provided that the system is persistent. Figure 3 shows five different runs with the low *D* – high *b* parameters, Supplementary Fig. S3 is the same for high *D* – high *b*. This underlines the repeatedly demonstrated^8,15^ robust nature of the MCRS, even with possible secondary structural – and thus also functional – differences between the complementary strands of the same RNA replicator. Furthermore, the changes in the lengths of the replicators show that the complete set of evolved RNA strands falls within the 35–45 nt range, and they are synchronized independently on the coded enzyme types.

This fact also emphasizes the importance and the effectiveness of the local group selection mechanism that keeps the system coexistent. Another aspect of the exceptional robustness of the system is that in the qualitative sense it is surprisingly insensitive even to replicator mobility (*D*) and length penalty (*b*_2_): we do not find conspicuous differences between the four parameter sets in this respect. Compare Fig. 3 and Supplementary Fig. S3, the first with high *D* – high *b*, the second with low *D* – high *b*. The two other combinations (not shown) give similar results. Some of the subtle quantitative differences will be touched upon in the Discussion.

### Evolution: optimization; parasitism vs. metabolism; emerging promiscuity

Mutations during replication may delete, insert or substitute nucleotides, thus potentially changing the length and/or the nucleotide sequence of the copy compared to the template strand. The daughter sequence is the complement of the mother if no mutation occurs, obviously resulting in a different secondary (folded) structure with the rare exception of the mother being a palindrome. The consequential constraint of any replicator probably returning to the same secondary structure in just every second generation is a strict one on the MCRS, because the system requires that all the metabolically necessary enzyme activities be present within the same metabolic neighbourhood for replication to occur at all. We expect there to be strong selective forces shaping the evolution of the replicators to multiply as fast as possible while maintaining as efficient a local metabolism as possible.

**Figure 4:**
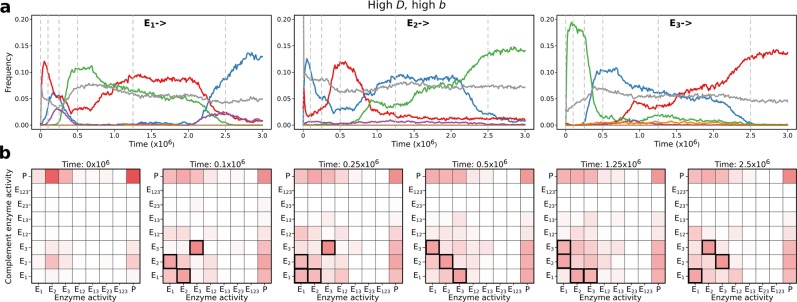
Dynamics of ribozyme activity distribution on complementary strands. **(a)** The temporal change in the frequency of all complementary pairs of monoactive ribozymes (*E*_*i* →_). The complementary strands may be *E*_1_ (blue), *E*_2_ (red), or *E*_3_ (green) ribozymes or parasites (grey). **(b)** Snapshots of the distribution of complementary pairs of RNA strands at the time points indicated by vertical dashed lines on **(a)**. Complementary pairs are identified by their enzymatic activities; darker shades of red represent more frequent activity pairs. Dominant activity pairs are denoted by black frames. Parameters: *D* = 4.0, *b*_2_ = 0.01 (high *D* – high *b*).

How the system deals with this optimization problem through mutations and selection can be seen by following the distributions of enzymatic activity patterns (*E*_*i*_ ↔ *E*_*j*_ distributions) for all the evolved complementary pairs of replicator strands within the replicator population^29^. Specifically, the frequencies of sequences harbouring any subset of the three different enzyme activities (*E*_1_, *E*_2_ and *E*_3_) were recorded for all the pairs of complementary replicator strands present in the lattice in all generations (Fig. 4). Note that any sequence with no metabolically active site (*P*) is a parasite lacking a metabolic functionality; the ones with a single activity on one of the strands (*E*_*i*_ ↔ *P*) are single-activity, whereas those with more than one active centres are *cis*-promiscuous strands (with, for instance, two active centres: *E*_*ii*_ homo- or *E*_*ij*_ heteroactive, depending on whether the same-strand active centres belong to the same activity type). A replicator sequence (i.e., a pair of complementary strands) is *trans*-promiscuous if both of its strands are enzymatically active, either with identical (homoactive) or with different (heteroactive) catalytic centres on the two strands.

The majority of the evolved sequences are *trans*-promiscuous ribozymes, single-activity ribozymes or parasitic sequences without any catalytic activity, almost irrespective of the actual model parameters. The frequency of *cis*-promiscuous enzymes is less than 1% (their scarcity represented by very light colours on Fig. 4b and Supplementary Fig. S5), which is why they are not shown in Fig. 4a. The typical absence of *cis*-promiscuity is easy to understand by considering that cramming more than a single catalytic motif into a short sequence is heavily punished by impaired functionality at all active sites of the molecule due to steric constraints (cf. Supplementary Equation (S2)). Even low replicator mobility (*D* = 1.0) is not sufficient to compensate for this effect and to select for substantial frequencies of *cis*-promiscuous strands in the system, see Supplementary Fig. S6.

Supplementary Fig. S6 shows the ribozyme activity distributions of the complementary pairs after 2.5 million generations for five replicate runs of the simulation at two different values of the length penalty parameter *b*_2_. In general, the vast majority of the replicator population consists of all kinds of single-activity (*E*_*i*_ ↔ *P*), monoactive *trans*-promiscuous (*E*_*i*_ ↔ *E*_*j*_) ribozymes, and parasites (*P* ↔ *P*). The parasitic sequences of each “quasi-species” are constantly produced by mutation from the corresponding catalytically active types. The two sets of simulations at high mobility (*D* = 4.0) with low (*b*_2_ = 0.005) and high (*b*_2_ = 0.01) length penalty slightly differ in their variation of activity patterns among the replicate runs: low length penalty allows for more variation due to higher scope for exploration within the sequence space, resulting in larger differences in their stationary activity patterns. The same difference is more conspicuous at lower replicator mobility (*D* = 1.0; see Supplementary Fig. S6).

An interesting aspect of the stability of the system is that the metabolic replicator populations evolve into single clusters or cluster pairs in sequence space (Supplementary Fig. S8), which form quasi-species in a loose sense of the term. At any point in time during the later phases of the simulations we observed such definite clusters and cluster pairs in the PCoA (Principal Coordinate Analysis) diagrams based on the Hamming distances of ten thousand sequences taken at random from the replicator community. The solitary clusters were dominated by nearly palindromic ribozymes, along with a substantial number of parasitic sequences present within the clusters. The cluster pairs tend to occupy two symmetric positions and take similar shapes in the space of the PCoA, and they were formed by trans-promiscuous ribozymes of complementary sequences which admitted different primary structures in every generation, and thus they alternated between the clusters of the pair. The two related clusters, together with the parasites mutated from them, constituted the quasi-species of the complementary pair of ribozymes. Supplementary Fig. S8 shows that parallel simulations tend to evolve into similar PCoA patterns, but this is not always the case. Regime shifts may reorganize the PCoA patterns just as they do the frequency distribution of catalytic activities (cf. Fig. 4 and Supplementary Fig. S5). Different parameter settings may result in seemingly different cluster patterns, but they preserve the quasi-species structure in all cases.

## Discussion

The dynamical feasibility criteria of origin-of-life scenarios require any prebiotic system to be stable in both the ecological and the evolutionary sense, while still remaining evolvable and capable of maintaining sequence diversity. We discuss the dynamics of the MCRS with regard to these fundamental criteria in the following sections, starting with the notion that a distinctive feature of the model is the low number of its parameters which, as well as the striking insensitivity of the dynamics to the actual parameter values, is a corollary of the fact that the dynamical parameters of the replicators are calculated from basic laws of physics and the feasible chemical consequences thereof – i.e., that they are determined by first principles. Some other model postulates are arbitrary and have just a very weak effect on the eco-evolutionary stability of the system (such arbitrary model features are the loop sizes and the sequence patterns of the replicator’s active sites), yet others (like diffusion rates, mutation rates, length penalty) can be changed over a wide range with a minimal dynamical effect because of surprisingly efficient stabilizing and regulatory mechanisms emergent from the first principles deployed.

### Ecological stability and robustness

Since the system is coexistent, the time-averages of the growth rates must be equal and at the stationary state they must precisely be equal to zero, otherwise one of the replicators would displace all the others (Fig. 3 and Supplementary Fig. S3) and the system would collapse. The equivalence of the average growth rates is enforced by the common regulation^30^ of the entire replicator community through the mutual dependence of the growth of any one replicator type on the presence of all the others due to their metabolic coupling.

The stabilizing effect of this regulatory mechanism is so efficient that the system remains robust under any reasonable parameter setting: it is highly insensitive to changing the values of its key parameters. The extreme stability of the sequence explicit MCRS is maintained by two direct and an indirect dynamical effect that RNA structure exerts on replicator fitness. The direct effects on the growth rates are realized through mutational changes affecting degradation and replicability, both of which are functions of folding energy and sequence length (cf. Supplementary Equations (S3) and (S5)), whereas the indirect effects come from the local metabolic support that neighbouring replicators provide for the local production of monomers. We consider the changes in replicator traits determining these direct and indirect effects in turn.

#### Direct effects on replicator fitness

**Figure 5:**
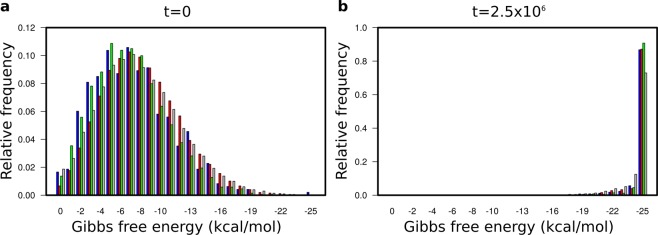
Distribution of Gibbs free energies. (**a**) The free energy distribution of the folded replicators *E*_1_ (blue)*, E*_2_ (red)*, E*_3_ (green) and *P* (grey) in the initial population (*t* = 0). (**b**) The evolved distribution at the end of the simulation (*t* = 2.5 10^6^). Note that the relative frequency scales are different on the two panels. Parameters: *D* = 4.0, *b*_2_ = 0.01 (high *D* – high *b*).

The free energy distribution of the molecules in an evolved system is steeply skewed towards the empirical minimum (which is about −25 kcal/mol in the permitted length interval; see Fig. 5 and Supplementary Fig. S4), so that the vast majority of persistent replicators are folded into a compact structure. Consequently, their degradation rates are also nearly uniform. As the lengths of the evolved strands follow a spiky distribution as well (in our specific case ranging from 35 to about 40 nucleotides in most simulations; cf. Fig. 3 and Supplementary Fig. S3), the replicability distribution of the evolved RNA population, which depends on folding energy and strand length, must be rather uniform as well. That is, the traits directly affecting replicator fitness (i.e., degradation rate and replicability) converge to the same value for all the different species of metabolic ribozymes due to their convergence towards the free energy minimum of the system.

#### Indirect effects on replicator fitness

Replicators have an indirect effect on their own fitness through contributing to the local metabolic efficiency of the system, i.e., through their enzymatic activities. Figure 3 and Supplementary Fig. S3 illustrates that the *E*_2_ and *E*_3_ activities converge to the same value, but *E*_1_ does not; *E*_1_ is 1.0 at all times. This is the only exception from the almost universal convergence in fitness-related properties, which, however, is the result of a hard arbitrary constraint enforced on the system: the *E*_1_ activity of any sequence is binary (0.0 or 1.0). This is yet another proof of its dynamical robustness: the system can withstand even such a crude arbitrary constraint. The evolutionary dynamics of the system sets the effective contribution of *E*_1_ to the common good equal to that of *E*_2_ and *E*_3_ by means other than tuning ribozyme activity, such as the relative “dilution” of *E*_1_ by allowing smaller but still substantial numbers of *E*_1_ ↔ *E*_2_ and *E*_1_ ↔ *E*_3_ replicators to persist (as *trans*-promiscuity, cf. Fig. 5a). That is, wherever a consistent trait convergence is prevented by a hard constraint, the system tunes other features by evolutionary means in order to compensate destabilizing effects. Notice that in this case compensation is possible precisely because the replicators exist in complementary pairs of sequences.

### Evolutionary stability and evolvability

#### Parasite resistance

A substantial fraction of the parasites of the system appear as the complements of metabolically functional replicators, which is why they cannot lose nucleotides to become shorter and thus to be copied faster: they have to yield functional ribozymes as their own copies if they are to survive the next few rounds of replication. Replicators with their complementary strands both parasitic are kept in check by their own adverse effect on the local metabolism wherever they become abundant. That is, the system does not allow highly destructive, short parasites with a slack secondary structure to persist and spread for long.

#### Evolutionary optimization

Both the ecological and the evolutionary stability of the MCRS are attained by evolutionary optimization acting on the complementary strands of each metabolic replicator simultaneously, but possibly aimed at different targets. The trivial target of optimization is replicability (*R*) as it directly contributes to the fitness of the replicators. This evolutionary pressure tends to decrease replicator lengths and increase their free energy so that they become short and loosely folded, i.e., it tempts the replicators to become parasites. An indirect pressure acts in the opposite direction to evolve them into efficient metabolic enzymes in order to make them capable of supplying themselves with monomers for their own replication by contributing to an efficient local metabolism. The actual lengths and folding energies of the strands are the results of a compromise between the evolutionary responses to these counteracting selective pressures: the optimal strands are long and folded into a sufficiently compact secondary structure to be good enough ribozymes, but short and loose enough to be copied relatively fast. In addition, the individual optimizations of all metabolic replicator species must to be balanced in such a way that neither ribozyme species excludes any other. The evolutionary dynamics of the sequence-explicit MCRS seems to be highly efficient in finding solutions to this delicate optimization problem. Although the solutions obtained in different parallel simulations with the same parameter set may differ in some detail such as the finer structure and the actual regime shift pattern of the evolved replicator community (cf. Fig. 4 and Supplementary Fig. S5), the diversity and the ecological and evolutionary stability properties of the resulting systems are always the same. The evolved convergence of the dynamical traits of the constituent replicators renders the MCRS exceptionally robust.

#### Emergent stability

One of the most important properties of the sequence-explicit MCRS model is that it does not require parametric fine-tuning to be persistent, evolutionarily stable and evolvable^28^. Most of the dynamical parameters of the replicators are derived from their nucleotide sequence and the corresponding secondary structural (folding) properties, which are in turn calculated from the physical-chemical characteristics of polymerized RNA nucleotides on a statistical physical basis. This is why a few dynamically straightforward parameters can define the replicability, degradation rate and enzymatic functionality of the replicators, along with all the well-known trade-off relations between them^21,22^. The dynamical parameters of the model are emergent from simple statistical physical rules, and they provide ecological stability to the system through the nearly infinite flexibility of the evolutionary process tuning them.

#### Regime shifts in activity pattern

The common features of the simulated activity patterns (Fig. 4 and Supplementary Figs. S5, S6) ultimately depend on the arbitrary choices of the catalytically active structures for *E*_1_, *E*_2_ and *E*_3_. We see that there is no unique fixed point in a typical simulation with a given set of parameters: in some cases the activity pattern may switch to a different one relatively fast. The different activity regimes can be well characterized by their different enzyme and complement enzyme activity patterns. All the replicator sets realized during the evolution of the system are metabolically complete at any point in time, but with different compositions under the different regimes. Note that these patterns seem to depend on the initial and actual active centre structures as well as on the model parameters. For example, *E*_1_ ↔ *E*_1_ is relatively rare if the mobility of the replicators is low (cf. Fig. 4 and Supplementary Figs. S5, S6), because highly localized replicators have a better chance of being metabolically complemented if their complementary strand carries a different metabolic function. In case of a more stringent length penalty (high *b*), bi-active ribozymes (i.e., *cis*-promiscuity) almost completely disappear (cf. Supplementary Fig. S6). The possibility of switching between different but in general equally viable states guarantees the adaptability of the system to external (environmental) fluctuations, and thus it is an important additional ecological and evolutionary stabilizing effect in the MCRS models. For a given parameter combination, the persistence time of the regimes is different for different ribozyme activity patterns, but the typical regime shift pattern is independent of the initial conditions. Unattained activity combinations (white cells in Supplementary Fig. S6) are energetically (or structurally) inaccessible to the system because of the length penalty and the actual choices of active centre structures: replicators mutating into these combinations are punished through group selection which is an essential coexistence mechanism in MCRS models.

#### Evolvability

The evolvability of the sequence-explicit MCRS is dependent on the availability of freely mutating replicators. This is a feature common for all versions of the model family^10^. Since parasites are always present, either in the form of both strands of the same replicator (*P* ↔ *P*) or in a ribozyme-parasite pair (*E*_*i*_ ↔ *P*), the possibility of them mutating into something useful for the system as a whole (such as new metabolic ribozymes, more efficient replicase ribozymes, or membrane-producing ribozymes) is always open. New functions (and the replicators providing them) have a chance to become integrated into a persistent system only if the disadvantage of increasing the system size is more than compensated for by the advantage of the services offered by the “recruit”. The easiest way of adopting new functions is through obtaining trans-promiscuous activities in *E*_*i*_ ↔ *P* type replicators, because it does not increase the number of mutualistic sequence pairs in the system. The dynamical consequences of this assumption are yet to be studied in later versions.

### Sustaining diversity: the error tolerance of the model

We have explored the error threshold of the MCRS using an empirical method, because analytical approximations are nearly impracticable in complicated agent-based lattice models like this one. For sake of simplicity, the effective mutation rate was considered to be the sum of the probabilities of substitution, insertion and deletion, see Supplementary Table S2. We have followed two scenarios in the simulations: *i)* the mutation rate was set to a fixed value at the beginning of the simulation (*non-adaptive scenario*); *ii)* all simulations were initiated by very low mutation rates (${p}_{sub}=0.005,\,{p}_{del}=0.0005,\,{p}_{ins}=0.0005$), and run until replicator properties converged (cf. Fig. 3), then the mutation rate was set to the same value as in the non-adaptive scenario (*pre-adaptive scenario*). Supplementary Fig. S7 shows the fraction of 35 independent replicate runs surviving 100.000 generations using the low *D* – low *b* parameter setting, as a function of the effective mutation rate. The pre-adaptive scenario allows for an error threshold more than twice as high as the non-adaptive scenario (*p = *0.066 vs. *p = *0.026). The difference is probably attributable to the fact that evolved systems are almost always much more stable than their ancestral systems, and thus they are also more resistant to mutational perturbation. Therefore, the error threshold of a pre-adaptive system may be considered as the upper bound for the error threshold of any possible non-adaptive system. Note, however, that even the non-adaptive version allows for almost three times as large a mutation rate than suggested by Eigen’s calculations^3^, mainly due to the fact that the phenotypic effect of a mutation event may be small with respect to catalytic activity, and even that effect remains local, possibly leading to the local extinction of the mutant if it is deleterious. The system collapses only if mutations affect too many localities simultaneously.

### A possible route towards chromosomes

Breaking the functional symmetry of the complementary strands^25^ could be a sensible way forward in prebiotic replicase evolution, since monoactive or *cis*-promiscuous replicators could have enjoyed the evolutionary advantage of a possible gene-enzyme specialization (high-energy unfolded “gene” and low-energy compact “enzyme”^31^). The prerequisite of such a transition is the presence of a sufficiently effective replicase ribozyme, enabling the “chromosomization” of separate metabolic replicators. A better replicase would speed up the replication process and improve its accuracy, which in turn would ease the selection pressure which keeps replicator lengths low. At the same time, cutting the functional (ribozyme) strand into separate enzymes requires the presence of at least one specific (restriction) endonuclease RNA^32,33^ as well. The hypothetical evolutionary scenario possibly leading to a feasible chromosomization in the MCRS is yet to be studied.

### Epilogue

As of today, there is no generally accepted scenario for the transition from chemical to biological evolution, i.e., for the emergence of “living matter”^34^ from the inorganic world. We think that the core idea of the MCRS could be a promising candidate for this role, as it offers a chemically plausible and biologically robust mechanism for this greatest of evolutionary transitions. As such, it may be a solid point of origin for further theoretical and experimental investigations into the process of the self-creation of life based on first principles.

## Supplementary information

Supplementary Information.

Supplementary Information.

## Data Availability

The datasets generated during and/or analysed during the current study are available from the corresponding author on request.
