# Supplementary material for: Dynamics and stability in prebiotic information integration: an RNA World model from first principles
Source: Sci Rep. 2020 Jan 9;10:51. doi: 10.1038/s41598-019-56986-8 (PMC6952369; doi:10.1038/s41598-019-56986-8)
Supplement: Supplementary file 2 — Supplementary Information. [file 41598_2019_56986_MOESM2_ESM.pdf]

SUPPLEMENTARY INFORMATION

Dynamics and stability in prebiotic information integration: an RNA World model from first principles

András Szilágyi, Balázs Könnyű and Tamás Czárán

Contents

Page

1. Details of the model **1**

1.1. Replicator properties: Catalytic activity, degradation, replication and mutation

1.2. Replicability vs. enzyme activity trade-off

1.3. Parasitic replicators

1.4. The initial RNA enzyme pool

2. Results of different runs **9**

2.1. Time series of parallel simulations with different parameters

2.2. Distribution of Gibbs free energy with different parameters

2.3. Dynamics of ribozyme activity distributions with different parameters

2.4. Activity distribution of ribozymes with different parameters

3. The error threshold **12**

4. Principal coordinate analysis **12**

5. Parameters of the model **13**

1. Details of the model

The replicators are RNA molecules represented by their nucleotide sequences, each sequence occurring in two different strands that are complementary to each other in accordance with the rules of nucleotide base pairing (A-U and G-C). The two most important properties of each strand of each replicator are their secondary (folded) structure and (Gibbs-) free energy (E) computed by the ViennaRNA Package [1] from their actual sequence. Notice that complementary pairs may be (and, most often, they are) different in these properties. Certain aspects of the primary and secondary structure are responsible for the catalytic activity of a given sequence, while free energy determines the stability of a given secondary structure, which in turn determines its degradation rate and also plays a role in its catalytic activity as explained below.

1.1. Replicator properties: Catalytic activity, degradation, replication and mutation

Catalytic activity. In the present version of the model we assume three different types of metabolically essential catalytic activities, each represented by predefined (arbitrary) motifs and structures of the folded RNA strands. The first one (E_1) is associated with a short nucleotide sequence string (motif) with no secondary structure; the other two (E_2 and E_3) are each associated with its own hairpin loop with predefined sequence motifs in the centre of the loop. In E_1 the level of the activity is binary: if the perfect motif is present in a linear region of the RNA (i.e., none of the bases of the motif are paired with any other base of the same strand), then the molecule is catalytically active, otherwise it is completely inactive. Specifically, we assume that a five-base-long sequence string ("GCGAU") is responsible for the first (E_1) activity. If this motif is present in any unstructured (linear) part of an RNA strand, then the first enzymatic activity is maximal, ($\alpha_1 = 1$), otherwise it is zero ($\alpha_1 = 0$).

The catalytic activities of the loops (E_2 and E_3) are graded: if a hairpin loop of the proper length is present, the accuracy of the motif forming the active site defines the level of the catalytic activity provided by the loop. The second catalytic activity (E_2) corresponds to an eight-base-long smaller loop, whereas the third one (E_3) corresponds to a larger loop consisting of 12 bases. The actual bases comprising the loop define the graded activity levels as follows:

- If two compulsory bases (Q) are present between two supporting bases (S) forming an ...SQQS... motif in the centre of the loop of the proper size, then the enzymatic activity is at its maximum, $\alpha_i = 1$ $(i = 2{,}3)$.
- If one of the two supporting bases is not the right one (X), so that the loop pattern is either ...SQQX... or ...XQQS..., then the activity falls to $\alpha_i = 0.9$ $(i = 2{,}3)$.
- If there are no supporting bases but both of the two compulsories are in the proper position ...XQQX..., the activity is $\alpha_i = 0.8$ $(i = 2{,}3)$.

- If there is only one compulsory base (no matter which one) in its correct position ...XXQX... or ...XQXX..., then the replicator attains a baseline activity of $\alpha_i = 0.1$ $(i = 2,3)$.

The two compulsory bases for the second activity (*E_2*) are G and G at loop positions 4 and 5, the two supporting bases are U and C at positions 3 and 6, respectively. For the third activity (*E_3*) the two compulsory bases are A and G at position 6 and 7, and the two supporting bases are G and A at 5 and 8. Table 1 summarizes the motif-to-activity correspondence for E_2 and E_3

**E_2 loop motif**	**E_3 loop motif**	**activity (α)**
((··**UGGC**··))	((····**GAGA**····))	1.0
((··X**GGC**··))	((····X**AGA**····))	0.9
((··**UGG**X··))	((····**GAG**X····))	0.9
((··X**GG**X··))	((····X**AG**X····))	0.8
((··X**G**XX··))	((····X**A**XX····))	0.1
((··XX**G**X··))	((····XX**G**X····))	0.1

Supplementary Table S1. Loop motifs and ribozyme activities for E_2 and E_3. X stands for any of the possible three alternative nucleotides at the corresponding nucleotide position of the optimal activity sequence. The standard dot-bracket notation has been used: matching parentheses and dots denote paired and free bases, respectively. The length of the helix (we indicate two matching pairs) does not affect activity. The proper compulsory and supporting bases are in boldface.

One sequence can harbour more than a single type of active site, in which case we call the ribozyme *promiscuous*. Promiscuity occurs in two different configurations in the model: either 1) one strand of a sequence is responsible for two or more different enzymatic activities (*cis-promiscuity*) or 2) one of the strands catalyses one reaction and its complementary strand catalyses another one (*trans-promiscuity*).

Cis-promiscuity is punished in this model with reduced activity for both active centres as it is expected to be, since an active centre almost inevitably constrains the functionality of the other one and vice versa, if they are on the same, relatively short molecule. In particular, for steric reasons, a *cis*-promiscuous enzyme cannot catalyse more than a single reaction at a time, thus the time sharing between different types of catalysis decreases both activities in a promiscuous ribozyme. We assume a sub-additive reduction by a factor of $\frac{1}{m^{\sigma}}$, where *m* is the number of activities present on the same RNA strand and $\sigma > 1$ ensures sub-additivity. This factor takes the time requirement of the induced enzyme-substrate fit into account, i.e., the time needed for the necessary conformational changes of the enzyme to take place for it to catalyse a reaction. Folding into the optimal conformation takes more time for *cis*-promiscuous enzymes because of their supposedly more rigid structures. Note that, due to

simple length or free energy constraints, very short sequences cannot be *cis*-promiscuous. In the (very rare) case of more of the same type of enzyme activity present within the same sequence, we use the average of the solitary activities.

Beyond the secondary structure, motifs of active sites and the number of different activities present, the catalytic activity is also affected by the free energy of the RNA molecule. The lower the free energy, the more stable the secondary structure of the molecule. For sake of simplicity, we assume that an RNA molecule can be in a folded secondary structure corresponding to the lowest level of free energy ($E < 0$) or a completely unfolded state with zero free energy ($E = 0$). Suboptimal secondary structures are completely ignored. In accordance with the principles of classical statistical mechanics, we assume that the probability of the system being in a state of energy E follows the Boltzmann distribution: the probability of being in a folded state of energy E is proportional to e^{-cE}: the probability of being in the unfolded state of energy $E = 0$ is proportional to 1. Thus the probability that a sequence is in a folded state (of free energy E) is

$$p_{\text{fold}} = 1 - \frac{1}{1+e^{-cE}} = \frac{e^{-cE}}{1+e^{-cE}} \quad \text{(S1)}$$

where c is a scale factor that guarantees that in the optimal energy state the folding probability $p_{\text{fold}} \approx 1$. The optimal energy state was approximated by folding 10^7 random sequences from the relevant length interval $L = 15 \ldots 75$. As the free energies within this population have an empirical lower bound at $E_{\min} = -25$ kcal/mol, we chose the constant $c = 0.3$. According to the resulting probability distribution a sequence with about zero free energy is in the folded state with probability ½, while a sequence with $E_{\min}$ with a probability 1.0.

Combining the sub-additive effect of multiple active sites and the distribution of folded-unfolded states the activity of an RNA molecule can $p_{\text{deg}} = \delta_0 - \delta_1$ be computed as

$$a_i = p_{\text{fold}} \frac{\alpha_i}{m^{\sigma}}. \quad \text{(S2)}$$

Note that *trans*-promiscuity is not actively punished in the model, but it conveys a temporal disadvantage: each generation of the *trans*-promiscuous enzyme can only catalyse one reaction, i.e., it behaves like a specialist enzyme at any time, but swaps activities in each generation.

Degradation. In each time step a replicator can replicate, remain idle, catalyse, or degrade. The probability of degradation depends on the free energy of the replicator according to

$$p_{\text{deg}} = \delta_0 - \delta_1 \frac{E}{E_{\min}}, \quad \text{(S3)}$$

where δ_0 is the degradation rate of a molecule in the zero free energy state (the maximum degradation rate) and δ_1 scales the stabilizing effect of lower free energy. E_{min} is the empirical minimum of the free energy of folded strains as previously introduced. In the present model we set $\delta_0 = 0.9$ and $\delta_1 = 0.8$. With this choice of degradation parameters the minimum degradation rate is 0.1, which prevents replicators from infinite persistence.

Replication. For a replication event to be successful a replicator *f* must be in the central site (focus) of a metabolic neighbourhood containing at least one active centre of each of the three different enzymatic activities (i.e., its neighbourhood must be metabolically complete). The three activities may be distributed on monoactive or promiscuous ribozymes: in the latter case, either in *cis* or in *trans* configuration. Let the metabolic neighbourhood of radius *h* around the focal individual *f* be denoted by $\Delta_h(f)$. The metabolic activity M_f around replicator *f* is the geometric mean of the metabolically essential enzyme activities within its metabolic neighbourhood (czaran2000):

$$M_f = \left[\prod_{i=1}^{3} \sum_{j \in \Delta_h(f)} a_{i,j} \right]^{\frac{1}{3}}. \tag{S4}$$

This function guarantees that, in the absence of one or more types of enzymatic activity (i.e., in an incomplete metabolic neighbourhood), the local metabolic activity around the focal replicator is zero: $M_f = 0$. Notice that for a given number of neighbours this function has its maximum at the possible *most even* distribution of the different enzyme functions within the metabolic neighbourhood.

In addition to the presence and distribution of all necessary ribozyme activities within the metabolic neighbourhood of the focal replicator the free energy and the length of *f* also affect its replication probability. Replication is possible only in the unfolded state; therefore, a factor $l + (1 - p_{\mathrm{fold}})$ is considered to affect replicability. $(1 - p_{\mathrm{fold}})$ is the probability of being in the unfolded state, and $l > 0$ ensures that even replicators at the lowest energy state, E_{min}, can replicate to some extent. The lengths also affect replicability in a linear way through two factors: one is responsible for the invariant part of replication time (length-independent initiation and termination step) denoted by b_1; the other is the part proportional to replicator length, the time needed to add a new nucleotide to the sequence being replicated, denoted by b_2 and multiplied by *L*. Introducing the scaling prefactor *g*, the length-dependent term is the following: $g\frac{1}{b_1+b_2L}$. These two factors together define the replicability (R_f) of a focal individual *f*:

$$R_f = g\frac{l + (1 - p_{\mathrm{fold}})}{b_1 + b_2 L}. \tag{S5}$$

Following [2] we define the claim C_f of replicator f belonging to the replication neighbourhood of an empty site to replicate into that empty site as the product of its metabolic support M_f, a function of its local monomer supply), and replicability (R_f):

$$C_f = M_f \cdot R_f \tag{S6}$$

The probability of the focal replicator f to replicate into an empty site in its replication neighbourhood is proportional to its share of all claims within the replication neighbourhood to occupy the empty site:

$$P_f = \frac{C_f}{C_e + \sum_j C_j}, \tag{S7}$$

where j runs across the replication neighbourhood of the empty site and C_e is the claim of an empty site to remain empty. We set $C_e = 1$. The probability that an empty site remains empty is

$$P_{\text{empty}} = 1 - \sum_j P_j. \tag{S8}$$

Mutation. The RNA molecule thus drawn for replication puts a complementary replica of itself onto the empty site of its own replication neighbourhood, with the nucleotide sequence of the copy determined by the classical Watson-Crick base pairing rules. Point mutations may take place during the replication process with per base probabilities p_{sub}, p_{ins}, and p_{del} , respectively resulting in base substitution, insertion or deletion. Either no mutation with probability $1 - (p_{\text{sub}} + p_{\text{ins}} + p_{\text{del}})$ or one of the three point mutation events may occur at each base copying step. Mutations at different positions of the sequence are independent.

1.2. Replicability vs. enzyme activity trade-off

The replicability and the metabolic activity of an RNA molecule are both dependent on its secondary structure, i.e., on the free energy state of the replicator. Since replication requires that the template be unfolded, whereas the metabolic function requires a more complex folded secondary structure, replicability and metabolic activity change in the opposite directions along the free energy scale, meaning that – in a stochastic manner – they are necessarily and automatically in a trade-off relation in this model. Low-energy sequences tend to be better enzymes but more difficult to copy, whereas high-energy sequences are easier to replicate but are likely to be less efficient metabolically. Note that some of the newer toy-model versions of MCRS incorporated replicability-to-metabolic-activity trade-offs by imposing explicit, reasonable, but still arbitrary trade-off functions on the system [3]. Here we do not need to define additional arbitrary parameters to prescribe the trade-off: it is an emergent property of the model.

1.3. Parasitic replicators

Replicator sequences with no enzymatic activity are parasites of the MCRS. They contribute nothing to metabolism that supplies monomers for replication: rather, they parasitize the local monomer pool produced by metabolically active RNA molecules nearby (cf. Fig. 2). Parasites are always present in the system: a substantial proportion of the initial replicator pool is parasites, and they are also continually produced from metabolic cooperators by mutation, generating a "quasi-species" for each ribozyme. They are even free to become shorter and form even less compact secondary structures, thus becoming easier to replicate and even more efficient as parasites. That is, they may evolve towards increasing their own replicability by both possible means: reducing their length and attaining an unfolded conformation.

1.4. The initial RNA enzyme pool

The initial spatial distribution of the replicator population was drawn from a ribozyme pool consisting of 6,5 million random RNA sequences generated within the 35–60 nt length range. All the sequences in the pool were folded by the ViennaRNA Package in order to calculate their minimum free energies in their folded states and to determine their enzymatic activities depending on the corresponding primary and secondary structures. A total of 344,208 of the sequences in the pool showed at least some enzymatic activity; the rest were metabolically inactive.

The simulations were initiated with RNA sequences drawn from this RNA pool using a layered random assignment algorithm. Since the frequency of enzymes is exceedingly low within the completely random sample of the initial ribozyme pool, complete local metabolic communities are scarce in initial states drawn from it at random. Therefore, at low initial concentrations of enzymatically active replicators the system collapsed rapidly in the overwhelming majority of attempts. To obtain viable systems at a sufficient frequency in the simulations, the relative proportion of metabolically active sequences had to be increased in the initial RNA population. This is why the layered algorithm of drawing initial sequences was applied: a fixed proportion of the initial population was drawn from the metabolically active part of the ribozyme pool, and the rest was chosen from the inactive (parasitic) sub-pool. We investigated the relationship between the initial proportion of enzymatically active replicators and the chance of system persistence. Supplementary Fig. S1 is a plot of the proportion of persistent systems against different active initial enzyme concentrations at low replicator mobility ($D = 1.0$). Exceedingly low initial densities of enzymatically active replicators hamper the assembly of complete local metabolic communities, and thus also the chance of system persistence. The condition for the system to be viable is that there be at least one complete metabolic neighbourhood within the lattice with all the required enzymatic activities present. This metabolically complete replicator assembly will then increase in size and spread until it invades the entire lattice. This means that the condition for the system to start up and attain its non-trivial steady state is to

initiate it on a sufficiently large lattice that has at least one such metabolic “seed”. At the limit of an infinite lattice, even an infinitely low initial concentration of metabolically active replicators would suffice for this to occur. The reason we are forced to use the augmented initial replicator population is that we cannot use very large lattices for lack of sufficient computing capacity. The actual lattice size, used in all simulations, was $300 \cdot 300$, which is a size close to the limit of practicability. Preliminary simulations were executed at both low (20%) and high (80%) initial ribozyme concentrations with this lattice size, showing that the stationary states of the persistent runs did not differ in the two cases. This suggests that the system is insensitive to the actual initial state as long as it allows it to take off. Therefore, we initiated all subsequent simulations at 1:1 ribozyme to parasite proportions.

Supplementary Fig. S2 summarizes the updating algorithm. This was repeated to each site of the lattice at a random order once on average in each generation, resulting in 90.000 elementary updates per unit time.

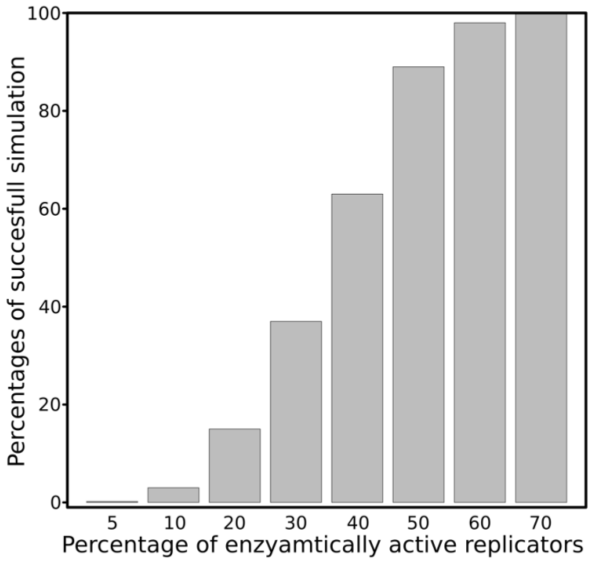

Supplementary Figure S1. The probability of system persistence at different initial ribozyme concentrations. Numbers are percentages of persistent outcomes from all simulations.

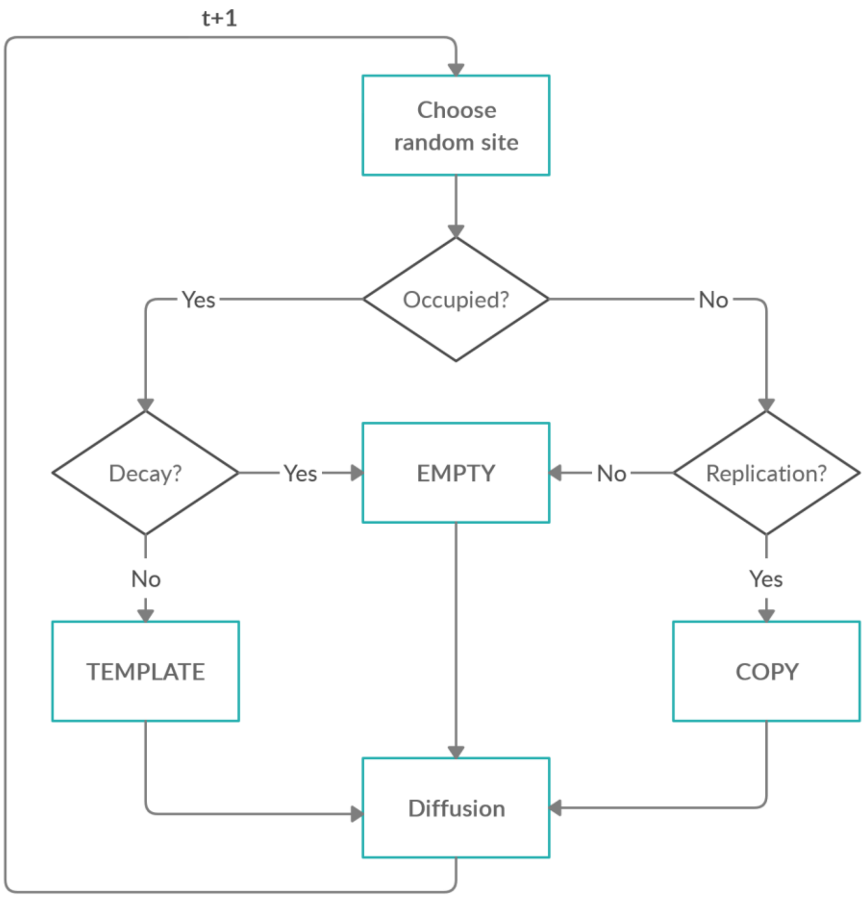

Supplementary Figure S2. Flow diagram of a single elementary step of the updating algorithm, repeated 90.000 times per unit time (generation). Simulations lasted up to $2.5 \cdot 10^6$.

2. Results of different runs

2.1. Time series of parallel simulations with the two parameter sets

In this section we show the results of simulations with different sets of parameters.

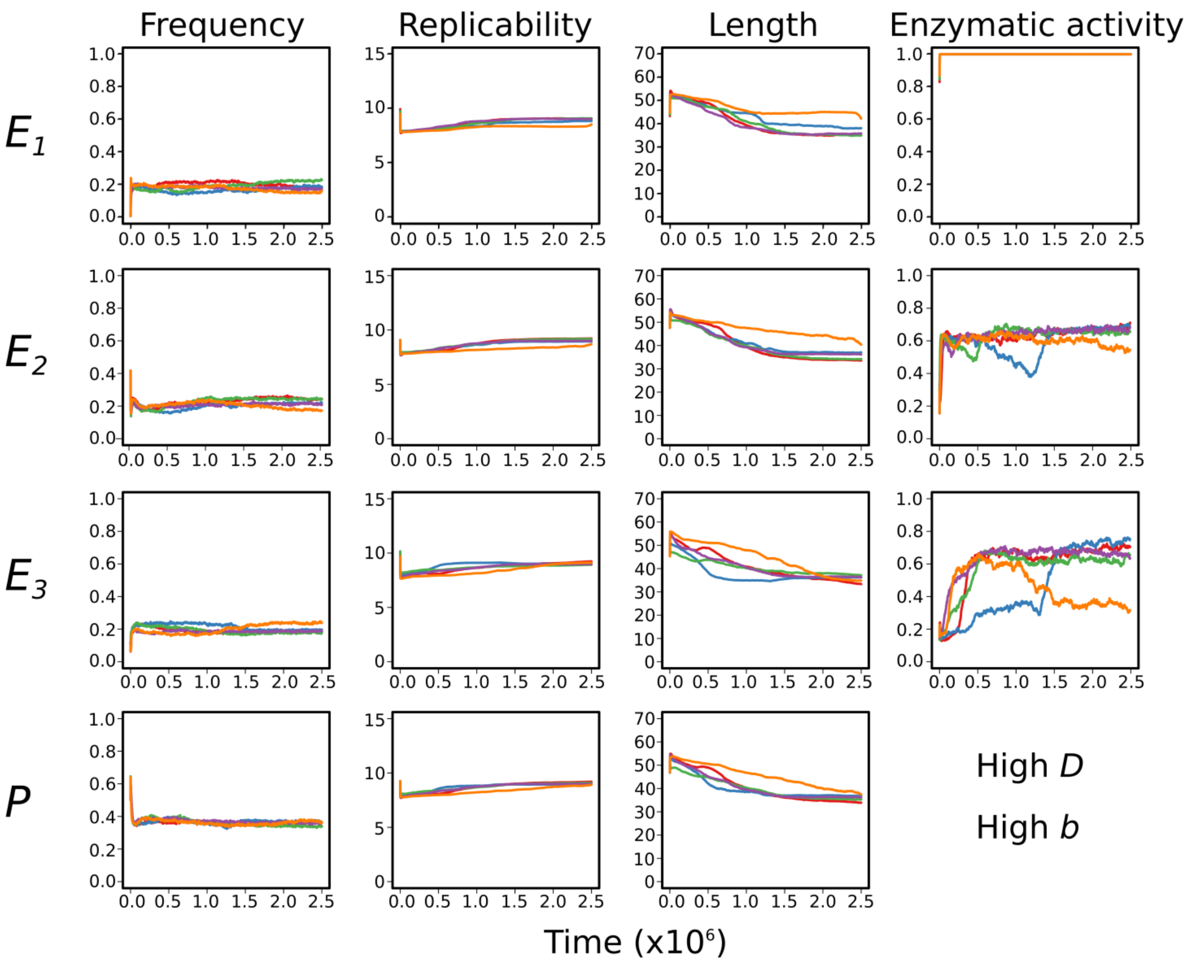

Supplementary Figure S3. Time series from parallel simulations. All plots show five replicate simulations with different runs in different colours. Replicate simulations were executed with identical parameter sets but different random number seeds and thus also with different initial RNA sequences. First column: frequencies of the three monoactive enzymes and functionless (parasitic) replicators; second, third and fourth columns: average replicabilities, lengths and enzymatic activities (based on Supplementary Equation (S5)), respectively. Initial proportion of enzymatically active replicators: 50%. Parameters: $D = 4.0$ and $b_2 = 0.01$ (high D – high b).

2.2. Distribution of Gibbs free energy with different parameters

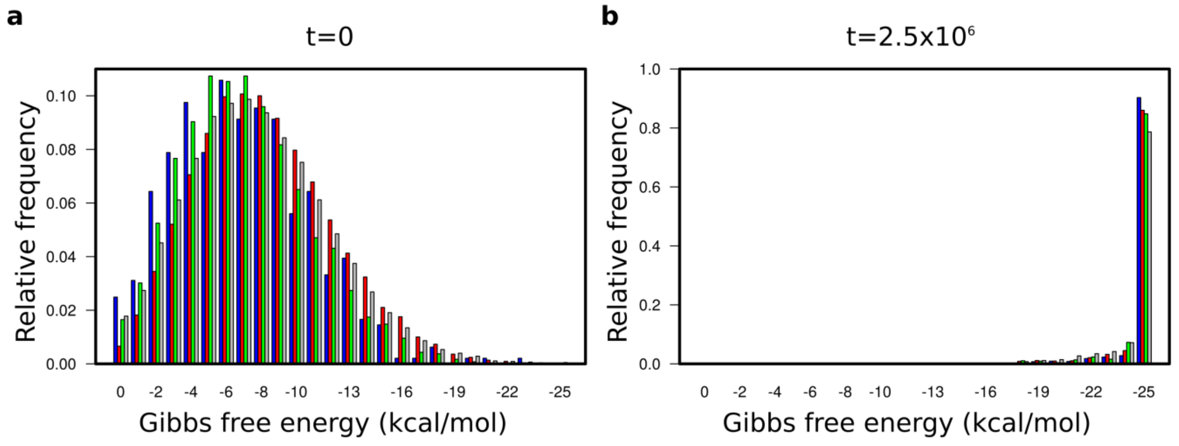

Supplementary Figure S4. Distribution of Gibbs free energies. **(a)** The free energy distribution of the folded replicators E_1 (blue), E_2 (red), E_3 (green) and P (grey) in the initial population (t = 0). **(b)** The evolved distribution at the end of the simulation ($t = 2.5 \cdot 10^6$). Note that the relative frequency scales are different on the two panels. Parameters are $D = 1.0$, and $b_2 = 0.01$ (low D – high b).

2.3. Dynamics of ribozyme activity distributions with different parameters

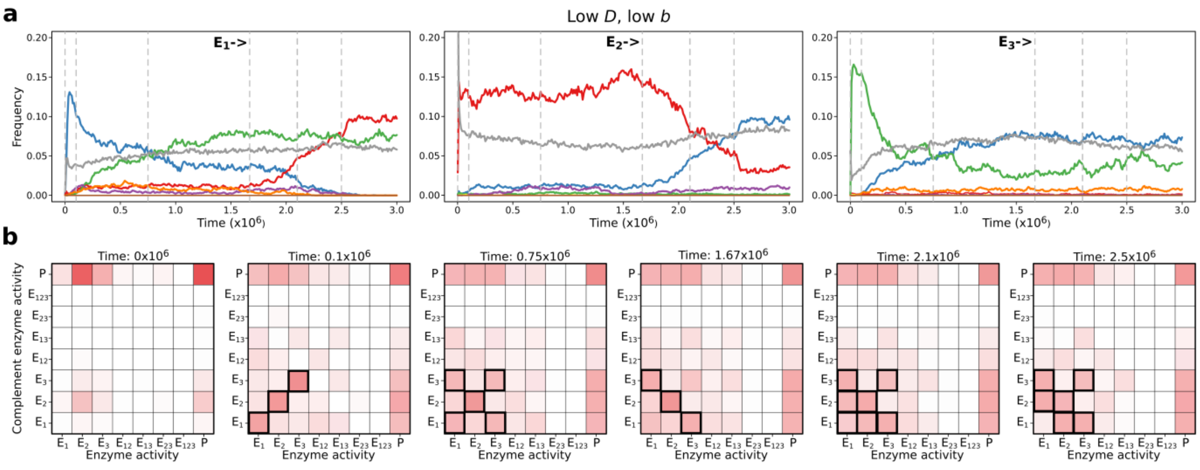

Supplementary Figure S5. Dynamics of ribozyme activity distribution on complementary strands. **(a)** The temporal change in frequency of all complementary pairs of monactive ribozymes ($E_i \rightarrow$). The complementary strands may be monoactive E_1 (blue), E_2 (red), or E_3 (green) ribozymes, biactive *cis*-promiscuous sequences – E_{12} (purple) and E_{13} (pink) – or parasites (grey). **(b)** The snapshots of the distribution of complementary pairs of RNA strands at the time points indicated by dashed lines on **(a)**. Complementary pairs are classified by their enzymatic activities at each time; darker shades of red represent more frequent activity pairs. Dominant activity pairs are denoted by black frames. Parameters: $D = 1.0$ and $b_2 = 0.005$ (low D – low b).

2.4. Activity distribution of ribozymes with different parameters

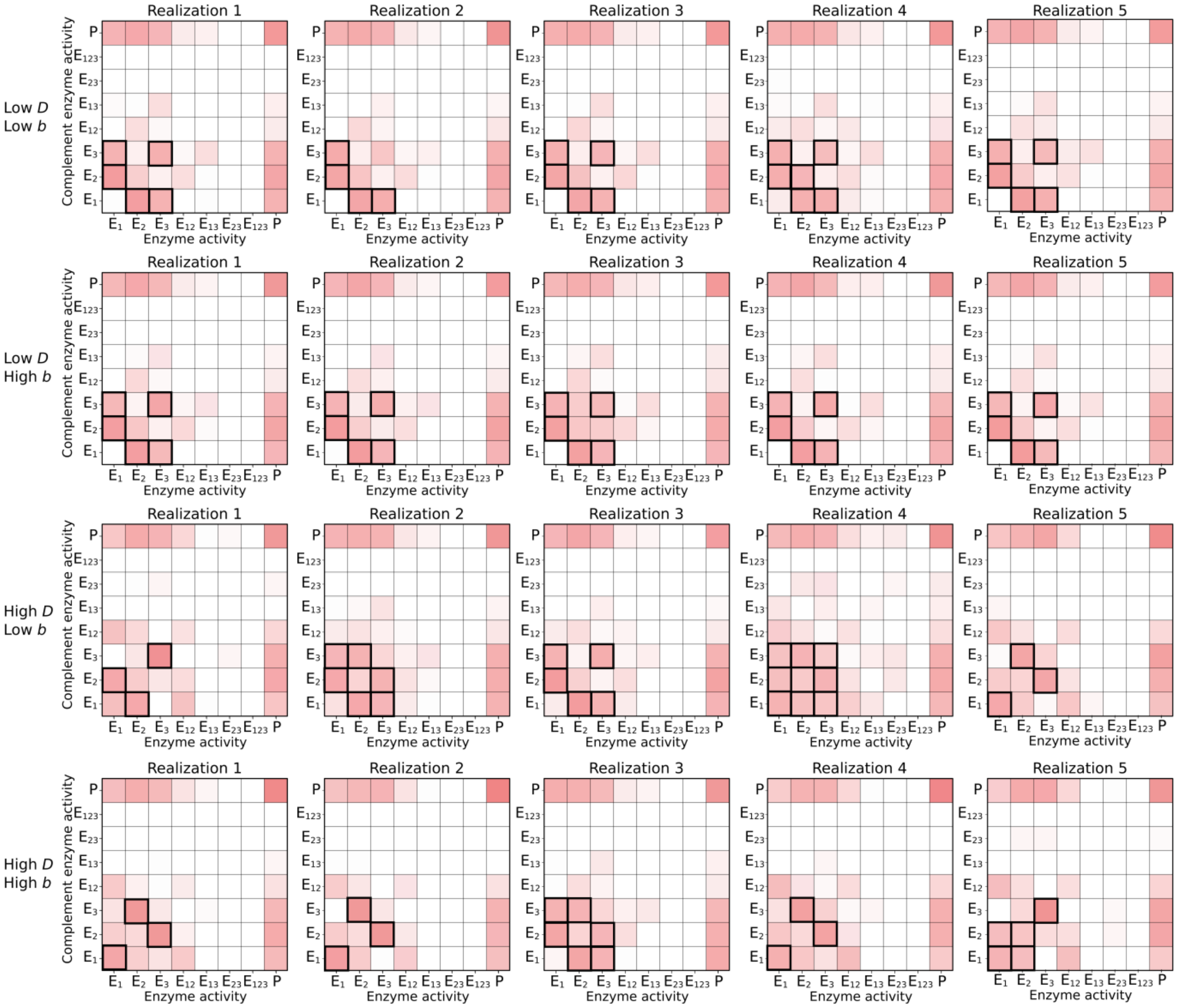

Supplementary Figure S6. Snapshots of the activity distribution of complementary RNA strand pairs in five replicate runs at four different parameter sets of the simulation. Panels show the distribution of the five parallel simulations at the final state ($2.5 \cdot 10^6$ th generation). Complementary pairs are classified by their enzymatic activities at each time point; darker shades of red represent more frequent activity pairs. Dominant activity pairs are denoted by black frames. The parameters for the four rows are: $D = 1.0$ and $b_2 = 0.005$ (low D – low b); $D = 1.0$ and $b_2 = 0.01$ (low D – high b); $D = 4.0$, and $b_2 = 0.005$ (high D – low b); $D = 4.0$, $b_2 = 0.01$ (high D – high b).

3. The error threshold

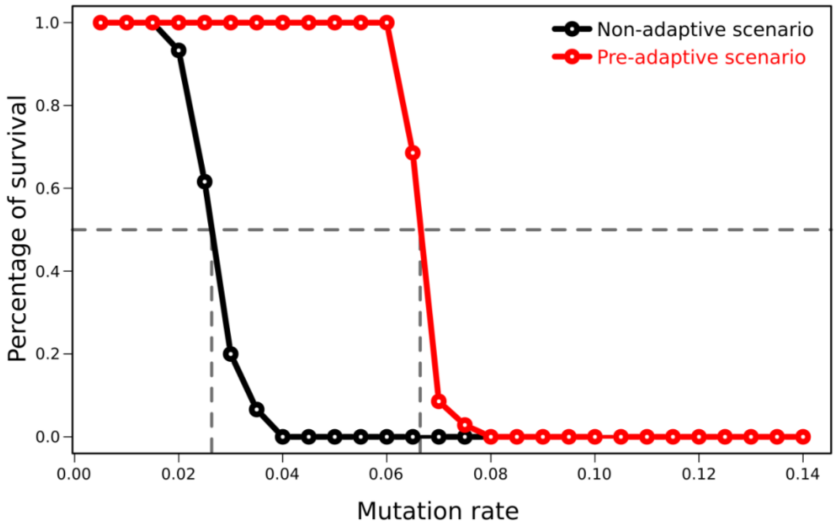

Supplementary Figure S7. The fraction of successful runs as a function of the mutation rate. The values are the average of 35 independent runs. The mutation rate is the sum of the per bit probability of substitution, insertion and deletion, cf. Table S2. Parameters: $D = 1.0$ and $b_2 = 0.005$ (low D – low b).

4. Principal coordinate analysis

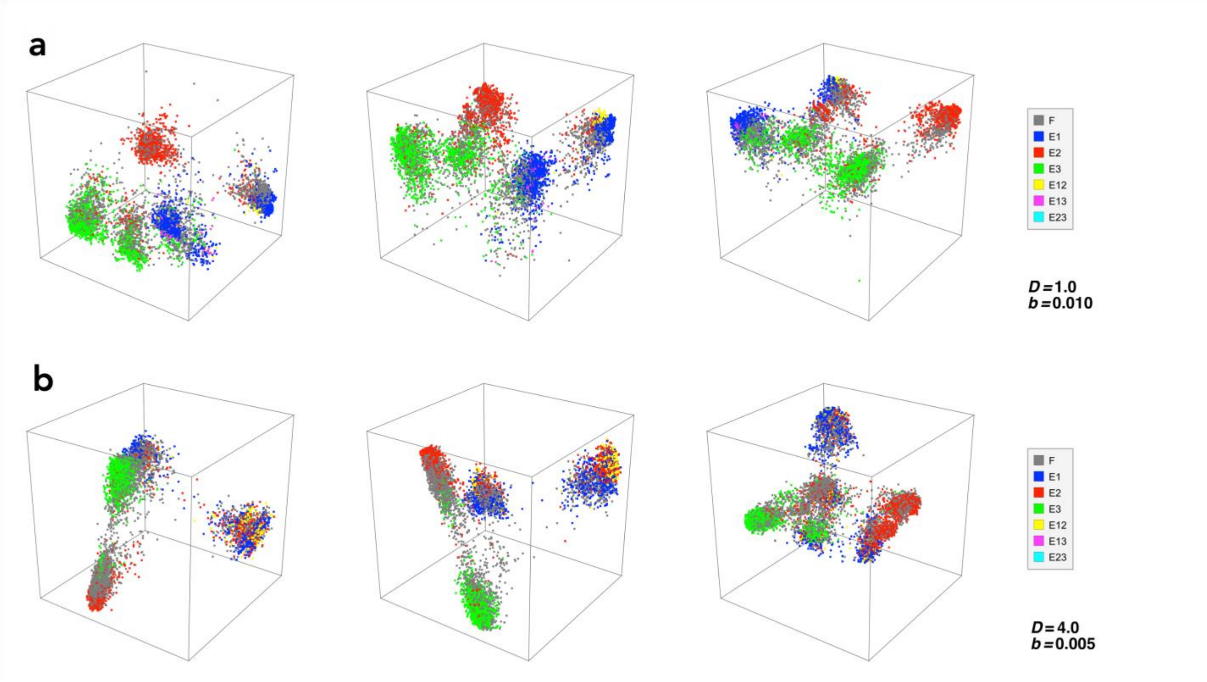

Supplementary Figure S8. Principal Coordinate Analysis (PCoA) results based on the Hamming distances between aligned replicator sequences. A random sample of 10.000 individual replicators with catalytic activity – E_1 (blue), E_2 (red), E_3 (green) , E_{12} (yellow) , E_{13} (magenta) and E_{23} (cyan) –

or without it, i.e., P (grey) in the evolved population (at $t = 3 \cdot 10^6$) are shown on each plot. Rows contain plots from three parallel simulations with identical parameters: in **(a)** $D = 1.0$ and $b_2 = 0.01$ (low D – high b); in **(b)** $D = 4.0$ and $b_2 = 0.005$ (high D – low b). For rotating PCoA plots with the parameters $D = 4.0$ and $b_2 = 0.005$ (high D – low b), see Supplementary Video V1.

5. Parameters of the model

Supplementary Table S2. Parameters of the model.

**Parameter**	**Description**	**Values**
$G \cdot G$	Grid size	300·300
$\boldsymbol{D}$	Number of steps in Toffoli-Margolus algorithm	1,4
h	Size of metabolic neighbourhood	9 (3·3, Moore-type)
r	Size of replication neighbourhood	5 (von Neumann type)
	Number of enzymes types	3
L	Maximum permitted length of replicators	70
C_e	Claim of an empty site to remain empty [a]	10%
c	Constant in Boltzmann-distribution	0.3
$\boldsymbol{p_{\mathrm{sub}}}$	Per base rate of substitution	0.005
$\boldsymbol{p_{\mathrm{ins}}}$	Per base rate of insertion	0.0005
$\boldsymbol{p_{\mathrm{del}}}$	Per base rate of deletion	0.0005
g	Scaling factor of replicability	10
σ	Penalty factor of *cis*-promiscuity	1.1
l	Additive part of $(1 - p_{\mathrm{fold}})$ in replicability	1
δ_0	Degradation rate if $E = 0$	0.9
δ_1	Energy dependent prefactor of degradation rate	0.8
b_1	Replication time (initiation)	1
$\boldsymbol{b_2}$	Per base elongation "length penalty"	0.01, 0.005

[a] Note that the claim of an empty site to remain empty (C_e) is defined as the 10% of its theoretical maximum. This maximum corresponds when all replicators with the highest activity present in the metabolic neighbourhood in the focal replicator and the focal one has the highest replication rate). Non-constant parameters marked in bold.

Supplementary Video V1. Rotating PCoA plots. Parameters are $D = 4.0$ and $b_2 = 0.005$ (high D – low b).

References

1 Lorenz, R. *et al.* ViennaRNA Package 2.0. *Algorithms for Molecular Biology* 6, 26 (2011).

2 Czárán, T. & Szathmáry, E. in *The Geometry of Ecological Interactions* (eds U. Dieckmann, R. Law, & J.A.J. Metz) 116–134 (Cambridge University Press, 2000).

3 Könnyű, B. & Czárán, T. The evolution of enzyme specificity in the metabolic replicator model of prebiotic evolution. *PLoS ONE* 6, e20931, doi:10.1371/journal.pone.0020931 (2011).
